# Antibodies as Tools for Characterization, Isolation and Production Enhancement of Anti-Cancer Drugs and Steroidal Hormones from Ginsenoside and Solasodine Glycoside: A Review

**DOI:** 10.3390/antib15010010

**Published:** 2026-01-19

**Authors:** Yukihiro Shoyama

**Affiliations:** Faculty of Pharmaceutical Sciences, Nagasaki International University, 2825-7 Huis Ten Bosch, Sasebo 859-3298, Nagasaki, Japan; shoyama@niu.ac.jp

**Keywords:** ginsenoside, solasodine glycoside, MAb, immunoaffinity concentration, scFv antibody

## Abstract

There are a vast number of monoclonal antibodies (MAbs) against biological components; however, the number for natural products is less than 50. MAbs against ginsenosides, i.e., dammarane triterpene glycosides contained in ginseng, were prepared to develop an Eastern blotting method that can estimate the number of bound sugars and pharmacological activity. Meanwhile, as a method for producing ginsenoside Rg3, which is used as an anti-cancer drug, an affinity column for ginsenoside Rb1 was prepared to isolate the raw material ginsenoside Rb1 in a single step, and a method for obtaining ginsenoside Rg3 through fermentation was proposed. A unique MAb capable of detecting all solasodine glycosides contained in *Solanum* plants was created to prepare an affinity column capable of isolating solasodine glycosides from *S. khasianum* fruit in a single step. The single-chain variable fragment gene was induced from the MAb against solasodine glycoside and introduced into the hairy root system of *S. khasianum*, thereby increasing the solasodine glycoside content more than twofold. As a result, we recognized that this method can be used to breed plants with higher concentrations of plant secondary metabolites like solasodine glycosides. The above results collectively demonstrate that solasodine glycoside can be isolated from *S. khasianum* in high yields and that this compound enables the production of steroids in high yields through a one-step chemical reaction.

## 1. Introduction

Since the dawn of human history, a vast number of plants have been used for medicinal purposes. Their uses can be roughly classified as follows: antibiotics [1], anti-cancer drugs [2], analgesics [3], immunosuppressants [4], heart disease treatments [5], and antimalaria drugs [6]. Therefore, approximately 50% of pharmaceuticals [7] are derived from natural sources, i.e., isolated or derived from plants, microorganisms, marine organisms, etc. Furthermore, many medicinal plants are also used in areas like traditional Chinese medicine, Kampo medicines, traditional Korean medicine, and Ayurveda.

For handling many types of medicinal plants, analytical methods for determining active ingredients and characteristic components are essential for maintaining a consistent quality of medicinal plants. The advent of thin-layer chromatography (TLC) and gas chromatography (GC) was followed by rapid developments in high-pressure liquid chromatography (HPLC) with MS (LC–MS) [8], LC–MS/MS [9], and UHPLC-QqQ-MS/MS [10], enabling high-sensitivity analysis. Monoclonal antibodies (MAbs) have been essential tools in biochemistry since the 1980s. MAbs were once limited to natural products such as morphine [11], but with the discovery of methods for determining the number of antigen molecules in haptens via MALDI-TOF MS [12], the number of MAbs has increased relative to the 1990s. The usefulness of MAbs against natural products has been recognized in a wide range of applications [13], including the confirmation of the activity of antigenic components in crude drugs, the evaluation of the quality of crude drugs and the in vivo dynamics of antigenic components, and the one-step isolation of antigenic components via an immunoaffinity column. Owing to their smallness, high tissue permeability, and the ease with which they can control blood concentrations, single-chain variable fragment (scFv) antibodies are being developed across a wide range of fields utilizing antibodies, including pharmaceuticals, particularly in cancer therapeutics and antibody–drug conjugates, diagnostics, sensors, and analytical kits [14]. However, the number of scFv antibodies targeting natural products is limited due to their restricted applications, as shown in Table 1 [15,16,17,18,19,20,21,22,23].

*Panax ginseng* C. A. Meyer is the most famous and important natural product with the name ginseng. It contains approximately 500 ginsenosides [24], as a major active constituent group, and polyacetylenes, sugars, amino acids, and alkaloids [25]. Ginseng has been well known since the Later Han period, and it has been used as an important drug in China. A total of 365 herbal medicines including animal- and mineral-derived crude drugs are listed in the Divine Farmer’s Classic of Materia Medica (100~200 AD) and are classified into three categories: highly safe (120 items), intermediate (120 items), and toxic (125 items). Ginseng, classified as highly safe, was said to have many mental benefits, such as healing the five organs, opening and calming the mind, stopping heart palpitations, eliminating evil spirits, enhancing cognition, and clarifying the eyes. Ginseng was introduced to Japan from China in the Nara period (710–794) during the reign of Shomu and has been included in the 60 Shosoin medicinal herbs.

In the early 1700s, a letter describing the natural habitat of ginseng in China was sent to Canada. Following expeditions made by Canadian people, American ginseng (*P. quinquefolius* Linne) was discovered, which was similar to the ginseng available in Canada in the 1710s. The overharvesting of wild species pushed it to the brink of extinction, but cultivation flourished in the 1800s. Today, approximately 1000 tons of dried American ginseng roots are produced and exported to China.

**Figure 1:**
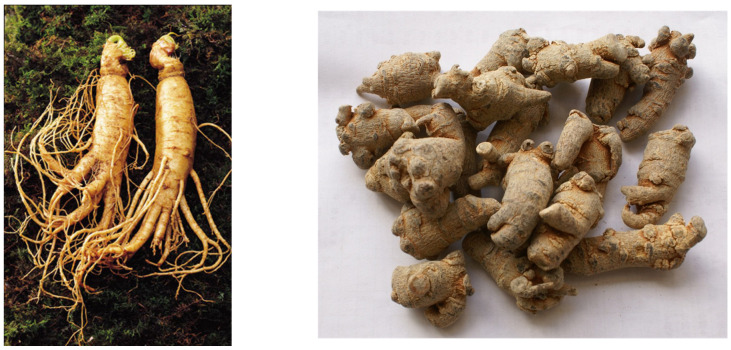
The shapes of ginseng roots: American ginseng (**left**) and Tanshichi ginseng (**right**) (photo by Y. Shoyama).

Tanshichi ginseng, *P. notoginseng* (Burk.) F.H. Chen, is a plant endemic to Yunnan Province in China. It was first discovered in the 16th century. The shape of the root is different from that of other *Panax* species (see Figure 1), and the medicinal properties described in the Honzo Tome [26] differ from those of ginseng; that is, it stops bleeding, disperses blood, and relieves pain.

**Figure 2:**
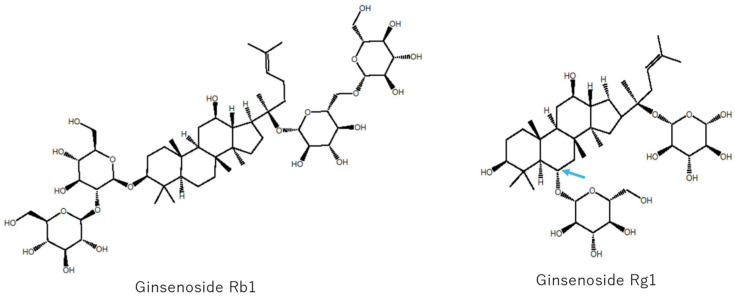
Structures of the major ginsenosides, ginsenoside Rb1 and ginsenoside Rg1, in *Panax* spp.; Arrow shows C-6 position.

*Panax* spp. have two types of structures, namely, protopanaxadiol-type ginsenoside Rb1 and protopanaxatriol-type ginsenoside Rg1, serving as major constituents, as indicated in Figure 2.

The aglycone formed when an OH group is attached to the C-6 position noted by the arrow is called the protopanaxatriol type. Protopanaxadiol-type and protopanaxatriol-type ginsenosides have different pharmacological effects. The former is known to have more powerful antitumor activity [27,28,29], while the latter is known to have strong anti-cognitive decline activity [30,31,32,33,34]. Protopanaxadiol-type ginsenosides have been reported to have strong antitumor activity; ginsenoside Rg3, which belongs to this category, is used in China as an anti-cancer agent, particularly as a metastasis prevention drug [35]. Ginsenoside Rd is also a protopanaxadiol-type ginsenoside like ginsenoside Rg3, indicating anti-cancer activity [36]. MAbs against ginsenosides such as ginsenoside Rb1 [37], ginsenoside Rg1 [38], ginsenoside Re [39], ginsenoside Rf [40], notoginsenoside R1 [41], ginsenoside Rg3 [42], ginsenoside Rh1, and ginsenoside Rg2 [43] have been prepared, and analytical methods using individual MAbs have been established. Alongside the advancement of antibody science, an MAb-staining method dubbed Eastern blotting [44] was established to determine pharmacological activity based on staining spots [45]. Fukuda et al. further prepared an affinity column loaded with an anti-ginsenoside Rb1 MAb and isolated ginsenoside Rb1 from ginseng extract in a single step [46]. The obtained ginsenoside Rb1 undergoes sequential cleavage of the C-20 sugar moiety through fermentation using *Microbacterium* spp. [47], yielding ginsenoside Rd and ginsenoside Rg3 in succession. Leveraging *Solanum* spp. in the family Solanaceae, which contains more than 2000 species, is the next task [48]. Among these species, *S. khasianum* C.B. Clarke is cultivated in India, China, Thailand, and other countries and used for medicinal purposes [49]. Solasodine glycosides are isolated from it and used for medicines, such as anti-inflammatory, anthelmintic [50], anti-diabetic [51], anti-acetylcholinesterase [52], anti-obesity, hepatoprotective, and anti-cancer drugs [53,54,55].

**Figure 3:**
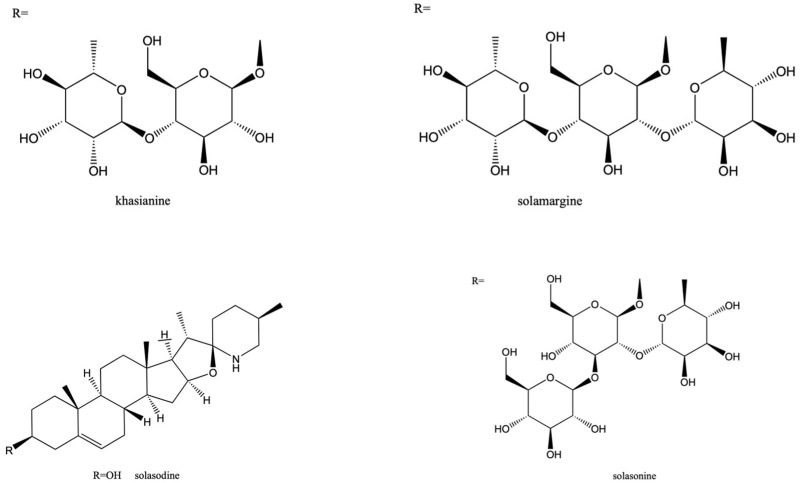
Structures of solasodine and its glycosides, khasianine, solamargine, and solasonine, contained in *Solanum khasianum*.

Pavani et al. reported the detection of steroidal alkaloids, flavonoids, phenols, tannins, steroids, and saponins in *S. khasianum* [56]. Mahato et al. isolated steroidal alkaloids such as solasonine, solamargine, and khasianine (Figure 3) from the fruit of *S. khasianum* and determined their structures via ^13^C NMR [57]. The antitumor activities of solasonine and solamargine have been confirmed using various cancer cell lines [58,59,60,61].

The structures of solasodine glycosides (Figure 3) and their pharmacological activities have been thoroughly studied, revealing their significance.

This review introduces methodologies using MAbs as an analytical tool, such as immunostaining (dubbed Eastern blotting) and a one-step purification method using an affinity column combined with MAbs specialized for the chemical structures of natural products like ginsenosides and solasodine glycosides. Furthermore, it describes a method for obtaining ginsenoside Rd and ginsenoside Rg3, which exhibit antitumor activity, through fermentation from ginsenoside Rb1 obtained from ginseng in a one-step process. For solasodine glycosides, we introduce the scFv gene for solamargine into *S. khasianum* cells to obtain plants with a high solasodine glycoside content. All solasodine glycosides are purified in a single step using an affinity column. We present a series of methods for obtaining steroid hormones from the resulting solasodine glycosides through chemical reactions.

## 2. Methods for Analyzing Natural Products Using MAbs

### 2.1. Eastern Blotting for Ginsenosides and Solasodine Glycosides

**Figure 4:**
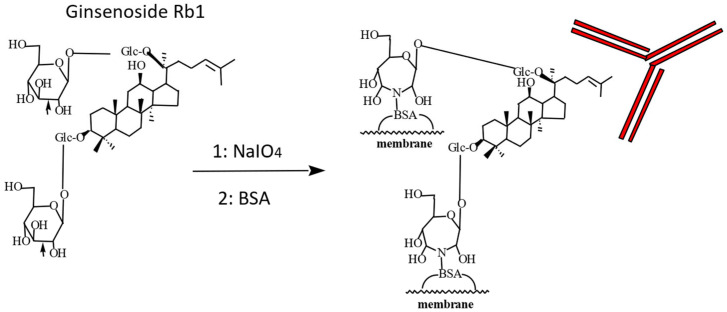
Scheme of using Eastern blotting to analyze ginsenoside Rb1.

A unique technique named Eastern blotting, a type of immunoblotting, is used to analyze ginsenoside Rb1. Ginsenoside Rb1, separated via TLC from ginseng extract, is transferred onto a membrane, such as PVDF, via heating. The membrane is treated with NaIO4 solution, yielding aldehyde groups. BSA solution is added to induce conjugation between the sugar moiety and BSA. The membrane-bound ginsenoside Rb1 is then visualized using its MAb in a manner similar to Western blotting, as shown in Figure 4.

**Figure 5:**
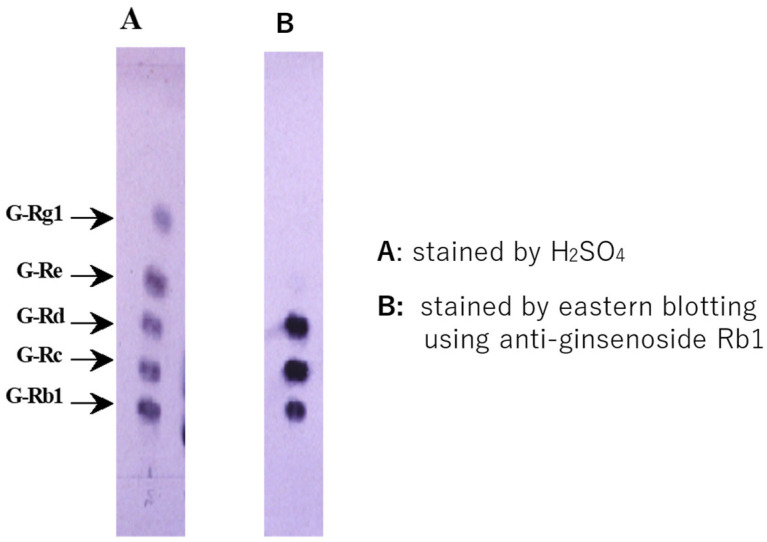
Eastern blotting profile of ginsenosides analyzed using anti-ginsenoside Rb1 Mab.

To stain ginsenosides via Eastern blotting, an anti-ginsenoside Rb1 MAb must be added to ginsenoside Rb1 immobilized on the membrane, as described above. The next step is to add labeled anti-IgG MAb followed by substrate to develop the color. Figure 5 shows the Eastern blotting analysis pathway for ginsenoside Rb1 [45].

Panel A in Figure 5 shows five ginsenoside standards developed via TLC and stained with H_2_SO_4_. All ginsenosides are stained. Meanwhile, panel B shows staining using Eastern blotting, where ginsenosides Rb1, Rc, and Rd are visible. These three ginsenosides belong to the protopanaxadiol category. This information demonstrates that using an MAb specific for protopanaxadiol-type ginsenosides allows for their detection [45]. Of course, protopanaxatriol-type ginsenosides Rg1 and Re can be stained using an anti-ginsenoside Rg1 MAb [45]. This can be performed by applying Eastern blotting to ginsenosides to assess their pharmacological activity. It is known that protopanaxadiol-type ginsenosides exhibit high antitumor activity [27,28,29] and anti-cognitive decline activity [30,31,32,33]. Additionally, double Eastern staining can be achieved by using both anti-ginsenoside Rb1 MAb and anti-ginsenoside Rg1 MAb, enabling the prediction of pharmacological activity for each spot [45]. Furthermore, from the Rf values in Figure 5, it can be inferred that the smaller the Rf value, the greater the number of sugar residues bound, depending on the type of sugar.

This section explains the Eastern blotting procedure for solasodine glycoside. The species *S. khasianum* primarily contains solasodine glycosides, though it also contains a few glycosides with other aglycones [57]. Furthermore, since the aim of this review was to synthesize steroid hormones from solasodine glycosides, the creation of an MAb with an affinity for all solasodine glycosides was required. Therefore, an MAb against solamargine was created, and it exhibited the broad affinity desired [62]. To investigate the characteristics of this MAb, we examined its cross-reactivity against various solasodine glycosides, yielding the following results: solamargine, 100%; solasonine, 92.1%; O-α-L-rhamnosyl-(1-2)-3-O-β-D-glucopyranosyl-solasodine, 36%; khasianine, 17%; and 3-O-β-D-glucopyranosyl-solasodine, 11% [62].

**Figure 6:**
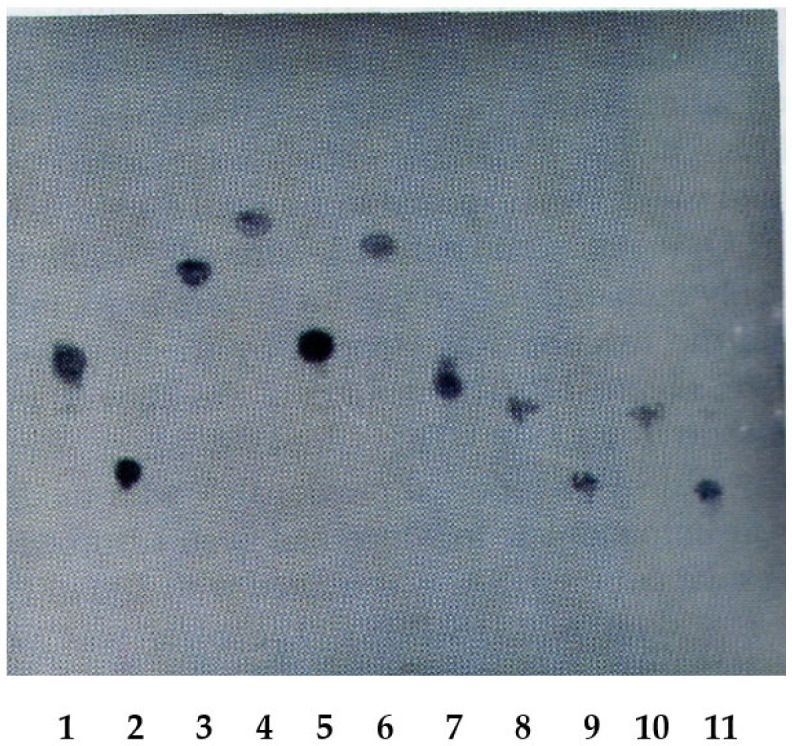
Easten blotting of solasodine glycosides using an anti-solamargine Mab. 1: Solamargine; 2: solasonine; 3: khasianine; 4: 3-O-β-D-glucopyranosyl solasodine; 5: O-α-L-rhamnosyl-(1-2)-3-O-β-D-glucopyranosyl solasodine; 6: 3-O-β-D-galactopyranosyl solasodine; 7: O-β-D-glucopyranosyl-(1-3)-3-O-β-D-galactopyranosyl solasodine; 8: solaverine I; 9: solaverine II; 10: 12-hydroxysolamargine; 11: 12-hydroxysolasonine.

To visually demonstrate this unique property, evidence from Eastern blotting that had been performed on solasodine glycoside was employed, revealing very interesting results; specifically, all solasodine glycosides were stained, as shown in Figure 6 [63]. Based on this evidence, the antibody was found to be fully suitable for the analysis of all solasodine glycosides in this study.

### 2.2. Preparation of a Single-Chain Fv MAb Against Solamargine and Its Application

**Figure 7:**
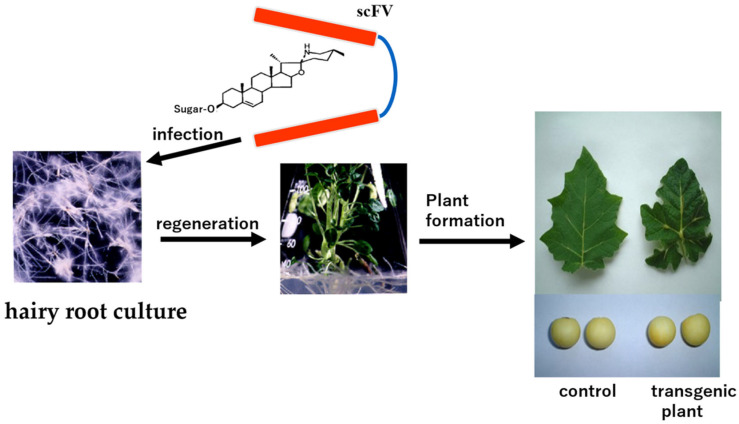
Preparation of scFv gene against solamargine and its use for increasing solasodine glycoside content in *S. khasianum*.

Putalun et al. developed recombinant antibody fragments, namely, scFv antibodies, against solamargine glycoside [20] derived from hybridoma cell lines [62]. The properties of the scFv protein expressed in *E. coli* were the same as those of the parent MAb [62]. It became evident that 220 ng of recombinant scFv was expressed in 1 mg of soluble protein of *S. khasianum* transgenic hairy root cultures obtained via infection. It is well known that Solanaceae plants possess a high capacity for redifferentiation from cells into plant bodies [64]. Therefore, when continuing the culturing of transgenic hairy roots, numerous hairy roots proliferated, underwent redifferentiation, and transitioned into plant bodies, as shown in Figure 7 [20]. Interestingly, there were 2.3-fold more solasodine glycosides, including solamargine, solasonine, and khasianine, in the transgenic hairy root than in the wild-type hairy root [20]. When the transgenic hairy roots were cultured in redifferentiation medium, redifferentiation occurred, leading to development into plant bodies that flowered and produced fruit. Since plant saponins are biosynthesized in phloem cells and stored in vacuoles [65,66], it is thought that the antigen–antibody reaction products—namely, the solasodine glycoside and scFv antibody complex, which may be an insoluble complex—are stored and accumulated in the vacuoles, resulting in approximately double the amount of solasodine glycoside biosynthesis. This fact provides important insights for plant breeding.

Regeneration from a transgenic hairy root occurred, yielding plantlets that grew into plants, bloomed, and bore fruit (see Figure 7). The concentration of solasodine glycoside in the fruits was about double that of the original plant [22]. The introduction of scFv genes into plant cells induces transformation, and plants with redifferentiation potential, like *S. khasianum*, can transform, making them applicable for breeding. As is well known, plant breeding involves cross-, mutation, polyploid, transgenic, genome-editing, and genomic breeding. The latest successful breeding method is a type of transgenic breeding. This novel method involves introducing scFv antibody genes and accordingly has been named “missile type molecule breeding.” [67]. Begum et al. collected many *S. khasianum* varieties and selected and bred varieties with high solasodine glycoside contents [68]. It may be possible to breed varieties with extremely high solasodine glycoside contents by infecting the good varieties selected in their study, i.e., those with high solasodine glycoside contents and transgenic hairy roots, and then re-differentiating the plants. The cited authors aimed to increase solasodine glycoside content, thereby improving the efficiency of steroid hormone production from solasodine glycosides. While prior research on plant-associated single-chain Fv antibodies successfully led to the creation of disease-resistant plants by introducing scFv targeting viral pathogens into host plants [69,70,71], this is the first example of applying scFv genes to breeding for low-molecular-weight natural products.

### 2.3. Purification via Immunoaffinity Column Conjugation of MAb for Ginsenosides and Solasodine Glycosides

**Figure 8:**
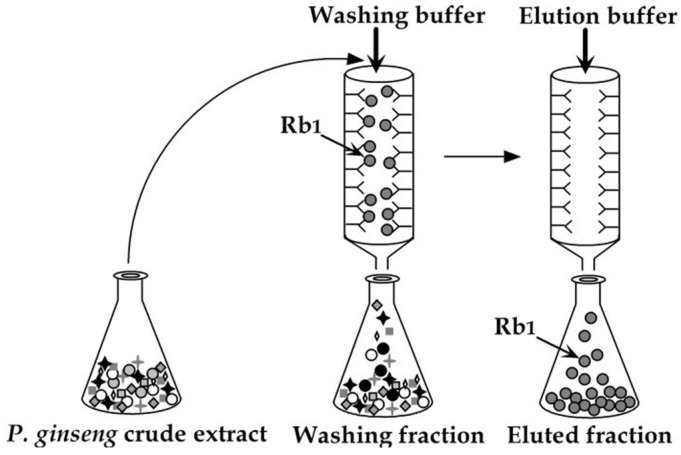
One-step purification of ginsenoside Rb1 using an immunoaffinity column combined with anti-ginsenoside Rb1 Mab.

The sugar chain of purified anti-ginsenoside MAb is oxidized and conjugated with hydrazide gel, yielding material for an immunoaffinity column. The crude extract of ginseng is charged to the immunoaffinity column and washed with phosphate buffer solution, yielding overcharged ginsenoside Rb1 and other components. After complete washing, ginsenoside Rb1 is eluted with AcOH buffer solution containing KSCN and MeOH to acquire pure ginsenoside Rb1(see Figure 8). The purity is confirmed via Eastern blotting, as discussed previously [46].

An immunoaffinity column for solasodine glycosides is prepared in the same way as in the case of ginsenoside Rb1. The *S. khasianum* fruit extract is loaded into an immunoaffinity column, washed with buffer solution, and then eluted with 40% MeOH in PBS solution to acquire the solasodine glycoside fraction, as confirmed by Eastern blotting using anti-solamargine Mab; as a result, only solamargine, solasonine, and khasianine are detected [72].

It has been clarified that only ginsenoside Rb1 or solasodine glycosides can be obtained in a single column using an immunoaffinity column, paving the way for the manufacture of the anti-cancer drugs ginsenoside Rd and Rg1 via fermentation and steroidal hormone preparations using the aglycone solasodine as a raw material.

## 3. Preparation of the Anti-Cancer Drugs Ginsenosides Rd and Rg3 from Ginsenoside Rb1 via Fermentation

**Figure 9:**
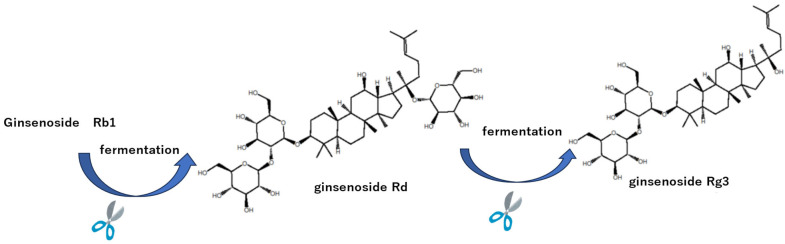
Production of the anti-cancer drugs ginsenoside Rd and Rg3 from ginsenoside Rb1.

Given that sugar chains cleave during steaming and/or fermentation, ginsenoside Rb1—which has long sugar chains and a high protopanaxadiol-type ginsenoside (the kind found in ginseng) content—was selected. As mentioned earlier, red ginseng contains ginsenosides, which are thought to be produced when one of the sugar chains is cleaved [73]. Unfortunately, quality control of red ginseng is considered difficult, so a different method must be selected. During fermentation, β-glucosidase secreted by lactic acid bacteria can hydrolyze major ginsenosides, such as ginsenosides Rb1 and Rg1, into minor ginsenosides, like Rg3, F2, and Rh2 [74,75]. Quan et al. isolated *Microbacterium esteraromaticum* from a sample from a ginseng farm. The 3β-glucosidase gene was cloned and expressed in *E. coli*. When ginsenoside Rb1 was incubated with the transgenic β-glucosidase, glucose at the C-20 position was cleaved in stages. That is, hydrolysis progressed from ginsenoside Rb1 to ginsenoside Rd and then ginsenoside Rg3 [47] as indicated in Figure 9. Ginsenoside Rd and Rg3 were purified from the fermented product via an affinity column loaded with ginsenoside Rg3 MAb [42].

## 4. Preparation of Steroid Hormone from Solasodine Glycosides

When synthesizing hormone agents using the natural product solasodine glycoside as a raw material, several steps are critical. 1. Solasodine glycoside must be obtained in a high yield. To achieve this, the scFv gene is introduced into *S. khasianum* plants to increase yield. 2. A method for rapidly isolating solasodine glycoside, which has a complex structural formula, must be developed. To achieve this, a unique immunoaffinity column must be created to develop a simplified isolation method. 3. Solasodine glycoside is hydrolyzed. 4. The resulting aglycone, solasodine, is converted into a steroid hormone via a chemical reaction. As outlined above, the raw material stage involves the use of MAb technology, and, ultimately, a one-step chemical reaction already under development is applied to produce the steroid hormone.

**Figure 10:**
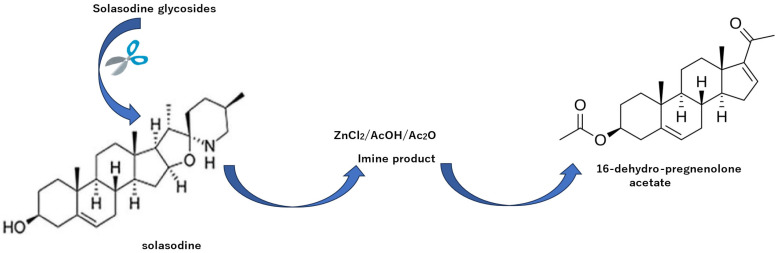
Preparation of steroid hormones from solasodine glycosides via a chemical reaction.

Figure 10 illustrates the step-wise conversion of solasodine into steroidal hormones. ZnCl_2_-AcOH causes the E and F rings of solasodine to open, producing an imine product; then, the double bond is cleaved to form a steroid structure, 16-dehydropregnenolone acetate [76].

## 5. Conclusions

**Figure 11:**
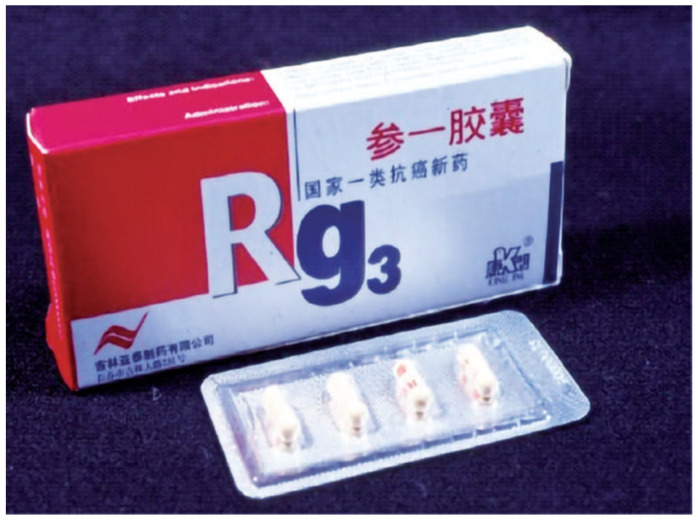
The anti-cancer drug ginsenoside Rg3 (photo by Y. Shoyama).

A total of 500 ginsenosides were isolated from ginseng and structurally elucidated [24]. They are divided into protopanaxadiol- and protopanaxatriol-type ginsenosides based on their aglycones, and they have different pharmacological activities. Therefore, MAbs against ginsenoside Rb1 and ginsenoside Rg1 were prepared, and an Eastern blotting method that can pharmacologically distinguish ginsenosides was developed. Additionally, an affinity column using an MAb against ginsenoside Rb1 was prepared, enabling the isolation of ginsenoside Rb1 in a single step. This makes it possible to produce ginsenoside Rg3, which is used as an anti-cancer agent in China (see Figure 11), from ginsenoside Rb1 through *β*-glucosidase [35].

The anti-solamargine MAb recognizes all solasodine glycosides, so it was successfully used to prepare an immunoaffinity column that can separate specific solasodine glycosides. Additionally, an anti-solamargine scFv antibody gene [20] was employed as a breeding method to increase solasodine glycoside concentrations, and this gene was introduced into *S. khasianum* via infection using a hairy root system, yielding a method for increasing solasodine glycoside content, thus opening the way for steroid hormone production using the aglycone solasodine as a raw material. These results may significantly contribute to steroid hormone production.

## Figures and Tables

**Table 1:** Preparation of single-chain Fv antibodies against natural products.

Plant Component	Plant Resource	Reference
Ganoderic acid	*Ganoderma lucidum*	[15]
Artemisinin	*Artemisia annua*	[16]
Paclitaxel	*Taxus brevifolia*	[17]
Harringtonine	*Cephalotaxus harringtonii*	[18]
Plumbagin	*Plumbago zeylanica*	[19]
Solamargine	*Solanum khasianum*	[20,22]
Morphine	*Papaver somuniterum*	[21]
Glycocholic acid	(Bile; animal)	[23]

## Data Availability

Data were taken from the references provided in the manuscript.
